# Use of non-insulin diabetes medicines after insulin initiation: A retrospective cohort study

**DOI:** 10.1371/journal.pone.0211820

**Published:** 2019-02-13

**Authors:** Yunwen Xu, Scott J. Pilla, G. Caleb Alexander, Irene B. Murimi

**Affiliations:** 1 Department of Epidemiology, Johns Hopkins Bloomberg School of Public Health, Baltimore, Maryland, United States of America; 2 Center for Drug Safety and Effectiveness, Johns Hopkins University, Baltimore, Maryland, United States of America; 3 Division of General Internal Medicine, Department of Medicine, Johns Hopkins Medicine, Baltimore, Maryland, United States of America; Mahidol University, THAILAND

## Abstract

**Background:**

Clinical guidelines recommend that metformin be continued after insulin is initiated among patients with type 2 diabetes, yet little is known regarding how often metformin or other non-insulin diabetes medications are continued in this setting.

**Methods:**

We conducted a retrospective cohort study to characterize rates and use patterns of six classes of non-insulin diabetes medications: biguanides (metformin), sulfonylureas, thiazolidinediones (TZDs), glucagon-like peptide 1 receptor agonists (GLP1 receptor agonists), dipeptidyl peptidase 4 inhibitors (DPP4 inhibitors), and sodium-glucose co-transporter inhibitors (SGLT2 inhibitors), among patients with type 2 diabetes initiating insulin. We used the 2010–2015 MarketScan Commercial Claims and Encounters data examining 72,971 patients with type 2 diabetes aged 18–65 years old who initiated insulin and had filled a prescription for a non-insulin diabetes medication in the 90 days prior to insulin initiation. Our primary outcome was the proportion of patients refilling the various non-insulin diabetes medications during the first 90 days after insulin initiation. We also used time-to-event analysis to characterize the time to discontinuation of specific medication classes.

**Results:**

Metformin was the most common non-insulin medication used prior to insulin initiation (N = 53,017, 72.7%), followed by sulfonylureas (N = 25,439, 34.9%) and DPP4 inhibitors (N = 8,540, 11.7%). More than four out of five patients (N = 65,902, 84.7%) refilled prescriptions for any non-insulin diabetes medications within 90 days after insulin initiation. Within that period, metformin remained the most common medication with the highest continuation rate of 84.6%, followed by SGLT2 inhibitors (81.9%) and TZDs (79.3%). Sulfonylureas were the least likely medications to be continued (73.6% continuation) though they remained the second most common medication class used after insulin initiation. The median time to discontinuation varied by therapeutic class from the longest time to discontinuation of 26.4 months among metformin users to the shortest (3.0 months) among SGLT2 inhibitor users.

**Conclusion:**

While metformin was commonly continued among commercially insured adults starting insulin, rates of continuation of other non-insulin diabetes medications were also high. Further studies are needed to determine the comparative effectiveness and safety of continuing insulin secretagogues and newer diabetes medications after insulin initiation.

## Background

Type 2 diabetes affects 30.3 million people in the United States and is a leading cause of morbidity and mortality. [1] Type 2 diabetes and its complications also pose enormous health and economic burdens, [2, 3] especially with respect to outpatient settings and prescription expenditures.[4] Given the progressive nature of type 2 diabetes, many patients eventually will be treated with insulin, which accounts for a large and increasing proportion of treatment expenditures.[5]

The transition to insulin is often a complex one. [6, 7] The American Diabetes Association (ADA) recommends continuing metformin therapy when insulin is started,[7] since metformin use has beneficial effects on reducing weight gain and insulin dose.[8] However, the relative benefits and harms of continuing non-insulin diabetes medications after insulin is started are unclear. ADA guidelines suggest that medications other than metformin may be discontinued on an individual basis, but professional guidelines do not include specific criteria by which this decision should be made. Notably, insulin secretagogues in combination with insulin may exacerbate weight gain and hypoglycemia, and there has been debate about whether these medications should be universally discontinued when insulin is started.[9–11] There is also little evidence as to whether newer diabetes medications, such as dipeptidyl peptidase 4 inhibitors (DPP4 inhibitors), glucagon-like peptide 1 receptor agonists (GLP1 receptor agonists) or sodium-glucose co-transporter inhibitors (SGLT2 inhibitors), should be continued after insulin is started.

Given these important therapeutic considerations, it is necessary to better understand practice patterns in diabetes treatment during the insulin transition. A previous study examined patterns of diabetes medication use during the insulin transition among participants in a large clinical trial.[12] This study found that within the first year of insulin initiation, metformin was continued among most patients (80.3%), and the majority of patients continued taking -combinations of two or more diabetes medications along with insulin.[13] This study was limited in that medication use was only assessed at yearly intervals, creating a question about how and when such medication changes are occurring. We identified no prior studies examining changes in diabetes treatment after starting insulin among the general U.S. population. Such studies are needed to determine whether diabetes treatment during the insulin transition is occurring in accordance with guidelines, and to identify where further research is needed to improve diabetes care during this critical period.

To fill this gap, we used a large dataset representative of commercially insured adults in the United States to conduct a retrospective cohort study characterizing patterns of non-insulin diabetes medication use among patients with type 2 diabetes as they transition to insulin therapy. To do so, we examined the prevalence of treatment continuation after insulin initiation, and used time-to-event analysis to characterize the time to discontinuation of specific therapeutic classes.

## Methods

### Setting

We used Truven Health Analytics’ Marketscan Commercial Claims and Encounters database for 2010–2015. The data is derived from varied health care providers across sites and types, and updates individual-level, healthcare reimbursement data on over 100 million U.S. residents annually. Each record includes anonymized patient identifiers, patient demographics, and service dates associated with a given claim. Dispensed prescriptions are coded using National Drug Codes (NDC) while diagnoses are coded using International Classification of Diseases, Ninth Revision, Clinical Modification (ICD-9-CM) systems.

### Participants

The sample population consisted of active employees, early retirees and their dependents who were enrolled in commercial insurance plans. Medicare is the primary payer for the elderly U.S. population and therefore some of their prescriptions would not be included in the commercial plans database.^14,15^ To be eligible for inclusion, we required that a patient: (1) was aged at least 18 but under 65 years on the date of their first observed insulin prescription, hereafter index date; (2) filled an insulin prescription between April 2010 and September 2015; (3) be continuously enrolled in a commercial insurance plan for at least 90 days prior to the index date; and (4) filled at least one prescription for a non-insulin diabetes medicine in the 90 days prior to the index date. We categorized non-insulin diabetes medications based on six therapeutic classes: biguanides (metformin), sulfonylureas, thiazolidinediones (TZDs), glucagon-like peptide 1 receptor agonists (GLP1 receptor agonists), dipeptidyl peptidase 4 inhibitors (DPP4 inhibitors), and sodium-glucose co-transporter inhibitors (SGLT2 inhibitors) (See **S1 Table**). We further excluded patients with type 1 diabetes, which was defined by the presence of any related health care encounter (diagnosis ICD-9: 250.x1, 250.x3) in the 90 days prior to the index date, due to fundamentally different management of type 1 and type 2 diabetes. We followed 72,971 patients who met inclusion criteria from the index date until all non-insulin diabetes medications were discontinued, the end of enrollment, or December 31 2015, whichever came first. The analysis was further limited to patients who were continuously enrolled for 90 days after the index date (N = 65,902, 90.3%), since patients might provide no claims or encounters after the index date due to switching their current insurance or other reasons, which would result in an underestimation of the non-insulin medication continuation rate.

### Analysis

We defined a medication as continued if the patient had used it in the 90 days prior to index date and filled at least one prescription of it after insulin initiation. We defined a medication as discontinued if the patients had used it before the index date but had no drug on hand for 90 days or more after insulin initiation. Patients using more than one diabetes medication were allowed to contribute information to more than one class, where we assumed that a decision to stop one non-insulin medication was not related to the decision to stop another.

We used two measures to characterize patterns of non-insulin diabetes medications. First, we computed the proportion of patients who had filled at least one prescription for a diabetes medication class within 90 days after starting insulin. The proportion was a proxy measure for the immediate decision to continue on non-insulin medication during the insulin transition. Second, we analyzed the decision to discontinue a non-insulin medication by calculating the time until the patient had no medication of a given drug class on hand for 90 or more days. We aggregated records on a medication class level, and thus allowed for patients using more than one non-insulin medication to contribute information to more than one class; for a given drug class, the unit of analysis was the individual.

We described baseline demographic characteristics by means with standard deviation (SD) and proportions, and continuation of different drug classes at 90 days after insulin initiation by proportions. We estimated the median time to discontinuation in months with a 95% confidence interval (95% CI) by Kaplan-Meier curves for each drug class. All statistical analyses were performed using SAS 9.4 (SAS Institute, Cary, NC). Two-sided P≤0.05 was considered statistically significant.

We performed a series of sensitivity analyses to assess the robustness of our findings. First, as an alternative to defining continuation based solely on a prescription refill after insulin initiation, we also allowed patients with non-insulin medications on hand on the day of insulin initiation to be counted as continuers of therapy, irrespective of whether they went on to refill the medication. This expanded definition assumes that the patients completed their existing diabetes medications after the transition to insulin. Second, we applied a more stringent definition of medication continuation that required patients to refill at least two prescriptions for a given non-insulin diabetes medication class instead of one.

## Results

### Participant characteristics

**Fig 1:**
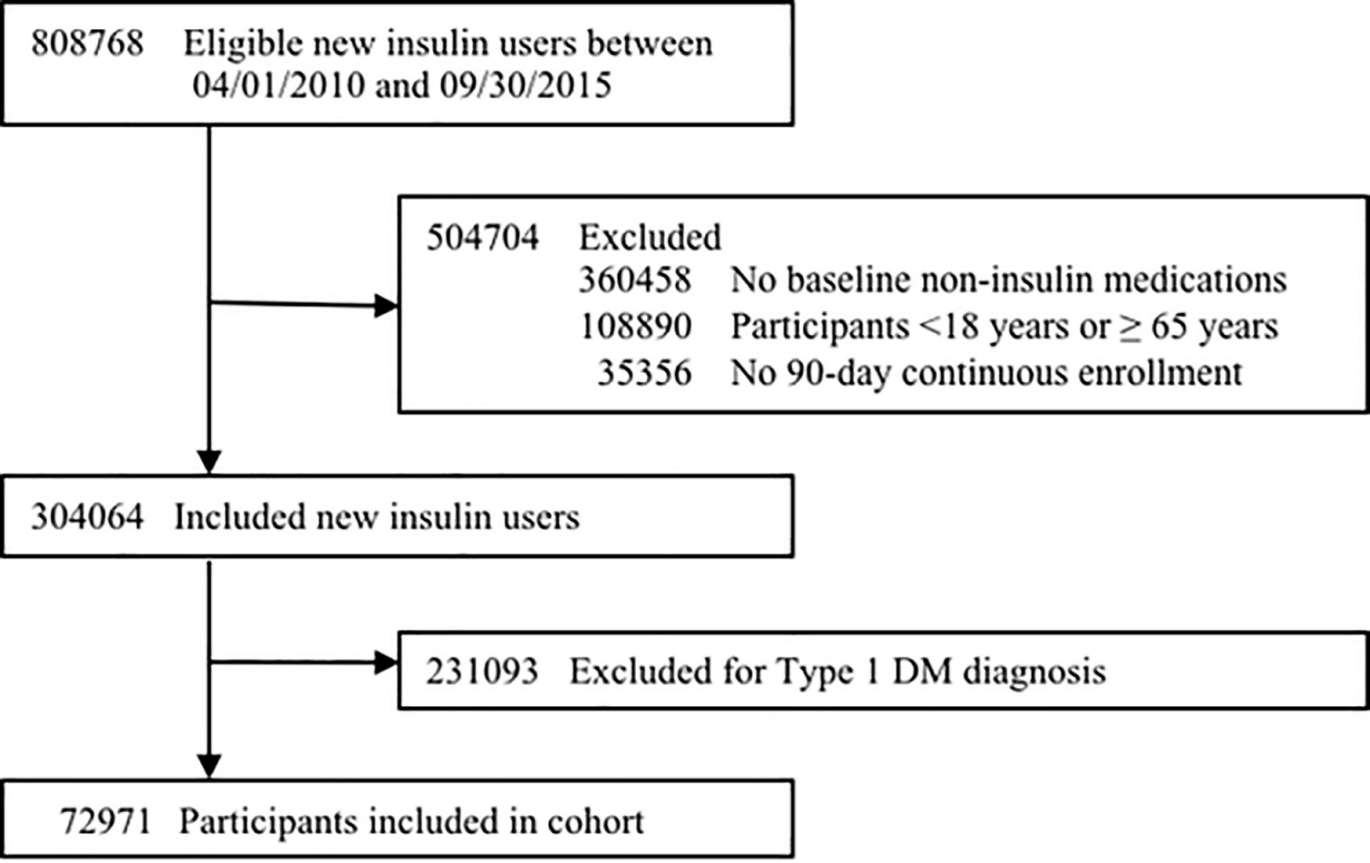
Flowchart of cohort creation process.

Out of 808,768 insulin initiators, 72,971 individuals were included in the final cohort (Fig 1). The mean follow-up was 13.3 months (SD = 12.4 months), with a median follow-up time of 9.7 months. Among included participants, the mean age was 51.5 years and 54.0% were male (Table 1).

**Table 1:** Baseline characteristics (N = 72,971).

Age, mean (SD), years	51.5 (9.6)
Male, n (%)	39,397 (54.0)
Baseline used diabetes medication classes, n (%)	
Metformin	53,017 (72.7)
Sulfonylurea	25,439 (34.9)
Dipeptidyl peptidase 4 inhibitor	8,540 (11.7)
Glucagon-like peptide-1 receptor agonist	6,074 (8.3)
Sodium glucose co-transporter inhibitor	1,133 (1.6)
Thiazolidinedione	7,461 (10.2)

### Diabetes treatments prior to insulin initiation

Approximately 7 of every 10 participants were on metformin before insulin initiation, and metformin was the most common therapeutic class (N = 53,017, 72.7%), while less than half of participants used other non-insulin diabetes medication classes. Followed by metformin, the most common classes used were sulfonylureas (N = 25,439, 34.9%), DPP4 inhibitors (N = 8,540, 11.7%), and TZDs (N = 7,461, 10.2%). There was low use of GLP-1 receptor agonists and SGLT-2 inhibitors which were used by 8.3% and 1.6% of participants, respectively (Table 1).

During the 90 days prior to starting insulin, participants used an average of 1.24 (SD = 0.54) non-insulin diabetes medications. Most participants (N = 54,072, 74.1%) received monotherapy, while the others used two or more diabetes medications, either as a fixed dose combination (e.g., metformin hydrochloride/saxagliptin hydrochloride) or two separate medications. Among those who used a fixed dose combination (N = 18,899, 25.9%), metformin plus sulfonylureas were the most common type (N = 9,691, 51.3%), while metformin was the most popular drug class compared to other single drug classes. (See S2 Table)

### Continuation of non-insulin diabetes medications after insulin initiation

**Fig 2:**
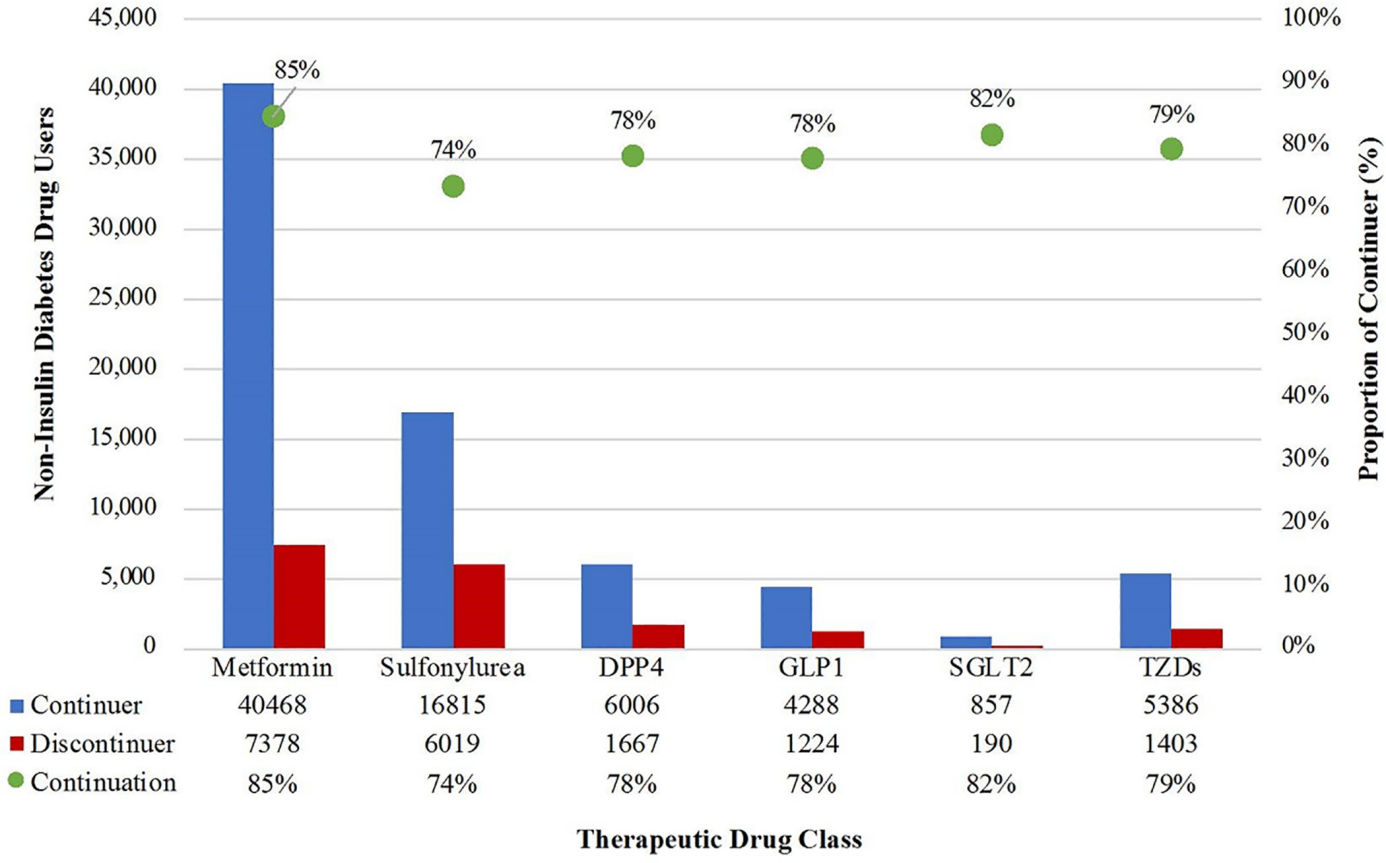
Non-insulin diabetes drug use after insulin initiation.

Out of the 65,902 individuals who remained insured for at least 90 days after the index date, 55,839 (84.7%) continued a non-insulin diabetes medication after insulin initiation. Rates of continuation varied across the six therapeutic classes (Table 2 and Fig 2). Metformin had the highest prevalence of use at roughly 73% both before and after insulin initiation, with the highest continuation proportion of 84.6%. DPP4 inhibitors and TZDs were similar both in terms of prevalence of use, roughly 11% and 10%, respectively, and rates of continuation, 78.3% and 79.3%, respectively. Although only 1.6% of patients were on SGLT2 before starting insulin, the continuation rate was 81.9%, a rate that was second only to metformin’s. On the other hand, sulfonylureas, which remained the second most commonly used diabetes medication class after insulin initiation, had the lowest rate of continuation at 73.6%.

**Table 2:** Non-insulin diabetes treatment use before and after insulin initiation.

Treatment Use	90 days Before	90 days After	Continuation (%) ^a^
**Overall, n**	65,902	55,839 ^b^	84.7
**Drug class, n (%)**			
Metformin	47,846 (72.6)	40,468 (72.5)	84.6
Sulfonylurea	22,834 (34.6)	16,815 (30.1)	73.6
Dipeptidyl peptidase 4 inhibitor	7,673 (11.6)	6,006 (10.8)	78.3
Glucagon-like peptide-1 receptor agonist	5,512 (8.4)	4,288 (7.7)	77.8
Sodium glucose co-transporter inhibitor	1,047 (1.6)	857 (1.5)	81.9
Thiazolidinedione	6,789 (10.3)	5,386 (9.6)	79.3

^a^: Continuation (%) was defined as the indicated medication class was used both before and after insulin initiation.

^b^: Among participants who were continuously enrolled for 90 days after initiating insulin (n = 65,902), the majority of them had refilled at least one non-insulin diabetes medication during the 90 days after insulin initiation (n = 55,839). Prevalence (%) after insulin initiation was defined as the proportion of participants used the indicated medication class among those who were continuously enrolled and used any non-insulin diabetes medications after insulin initiation (denominator n = 55,839).

Subsequently, we compared patients whose last filled prescription prior to insulin initiation was a single-compound non-insulin medication to those who were using multiple non-insulin diabetes medications. Comparisons of continuation rates between single-compound and multiple medication users are shown in Table 3. For both groups, metformin was the most commonly continued medication (83.4% for monotherapy users and 79.6% for combination therapy users) followed by SGLT2 inhibitors (79.2% and 77.2%, respectively). However, there were between-group differences in both overall and class-specific rates of continuation. Overall, a higher proportion of patients on combination medications continued the diabetes medications than patients that had been on non-insulin monotherapy at baseline (88.6% versus 80.0%, respectively). However, class specific continuation rates were higher for patients on single compounds. For example, GLP1 receptor agonists had the lowest continuation proportion of 76.4% among monotherapy users compared to 72.9% among those who used GLP1 receptor agonists in combination with other non-insulin diabetes medications.

**Table 3:** Proportion of patients continuing non-insulin diabetes medications after insulin initiation comparing baseline monotherapy versus combination therapy users.

Continuation ^a^ n/N (%)	Monotherapy users	Combination therapy users
**Overall**	**26,311 / 32,568 (80.8)**	**29,528 / 33,334 (88.6)**
**Drug class**		
Metformin	13,851 / 16,604 (83.4)	26,617 / 33,435 (79.6)
Sulfonylurea	6,640 / 8,464 (78.4)	10,175 / 15,813 (64.3)
Dipeptidyl peptidase 4 inhibitor	2,205 / 2,838 (77.7)	3,801 / 5,379 (70.7)
Glucagon-like peptide-1 receptor agonist	1,119 / 1,464 (76.4)	3,169 / 4,350 (72.9)
Sodium glucose co-transporter inhibitor	251 / 317 (79.2)	606 / 785 (77.2)
Thiazolidinedione	2,245 / 2,881 (77.9)	3,141 / 4,352 (72.2)

a:Continuation (%) was defined as a medication was used both before and after insulin initiation.

### Time-to-event analysis of discontinuation of non-insulin diabetes therapy

**Fig 3:**
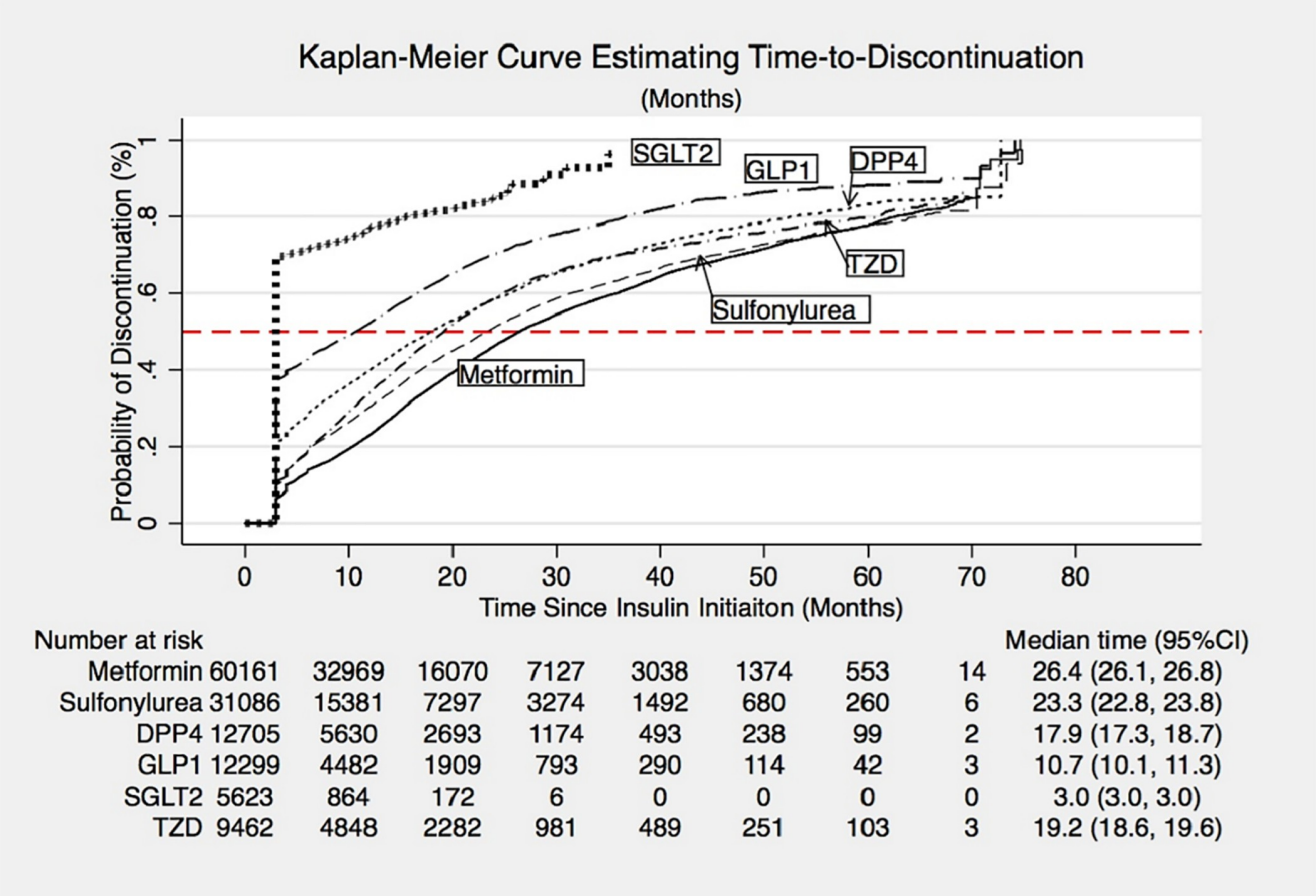
Kaplan-meier curves with risk set of time-to-discontinuation.

The class-specific median time to discontinuation differed across drug classes. (Fig 3). After insulin initiation, participants remained on metformin for the longest period of time (median of 26.4 months), followed by sulfonylureas (23.3 months) and thiazolidinediones (19.2 months). SGLT2 inhibitors and GLP1 receptor agonists were discontinued sooner after insulin initiation (Table 4), with a median time to discontinuation of 3.0 months and 10.7 months, respectively.

**Table 4:** Kaplan-meier estimates of median time-to-discontinuation (months).

Time-to-discontinuation	Cohort		Discontinuers*	
(months)	Median	95%CI	Median	95%CI
Drug class				
Metformin	26.4	(26.1, 26.8)	11.2	(11.0, 11.4)
Sulfonylurea	23.3	(22.8, 23.8)	8.0	(7.6, 8.2)
Dipeptidyl peptidase 4 inhibitor	17.9	(17.3, 18.7)	5.0	(4.8, 5.4)
Glucagon-like peptide-1 receptor agonist	10.7	(10.1, 11.3)	3.0	(3.0, 3.0)
Sodium glucose co-transporter inhibitor	3.0	(3.0, 3.0)	3.0	(3.0, 3.0)
Thiazolidinedione	19.2	(18.6, 19.6)	9.2	(8.8, 9.5)

*Analysis limited to patients who ended up discontinuing the treatment

### Sensitivity analyses

The results of our sensitivity analyses were generally consistent with the findings of the primary investigation (see S3 and S4 Tables). Namely, that majority of patients continued non-insulin diabetes medications after insulin initiation, with differences across drug classes in the proportion being continued but the level of continued use differs across therapeutic drug classes. For example, we identified 38,045 participants who had refilled at least two prescriptions for a given non-insulin diabetes medication class. Metformin and sulfonylureas remained the two most commonly used drug classes under this more stringent case definition after insulin initiation, at roughly around 70% and 30%, respectively. The overall continuation proportion dropped from 84.7% to 57.7%, and all class-specific ones were decreased by more than 20%. SGLT2 inhibitors had succeeded metformin to be the most continued drug class; while sulfonylureas were still the least continued drug class (S3 Table).

## Discussion

In this study, we used anonymized, administrative claims data from 2010–2015 to describe patterns of non-insulin diabetes medication use after insulin initiation among commercially insured U.S. adults. We found that in the first 90 days after insulin initiation, the vast majority of patients continued their previous non-insulin therapy, including 84.6% of patients continuing metformin and 78–82% of patients continuing newer diabetes medications. In addition, the vast majority of patients taking insulin secretagogues continued them after insulin initiation, with a median time to discontinuation of almost two years. As insulin becomes an increasingly common treatment for patients with type 2 diabetes, [5] these findings highlight the critical need for studies of the comparative effects of continuing versus stopping insulin secretagogues and newer diabetes medications after insulin is started.

The high proportion of patients continuing metformin therapy after insulin initiation and long duration of use after insulin initiation suggest that, in respect to metformin, clinicians are providing care in accordance with practice guidelines issued by the ADA.[7] This finding is also in agreement with prior studies that found a high proportion of metformin continuation and reported metformin to be the most commonly used therapy in combination with insulin.[12, 13] There is a wealth of evidence to support the benefits of continuing metformin in this setting, including its association with better glycemic control, lower weight gain and lower insulin doses. [8, 14, 15]

While metformin was largely continued after insulin initiation, it is important to consider the reasons why discontinuation may have occurred. Gastrointestinal disturbances are the most common side effect of metformin which affects 20–30% of patients and often results in discontinuation, though symptoms can be minimized or avoided by careful dose titration.[16] The major contraindication to metformin is renal impairment due to concerns of metformin causing lactic acidosis, though there is substantial evidence that the association between metformin and lactic acidosis is weak, and is predominantly a concern among patients with glomerular filtration rates less than 30 mL/min/1.73m^2^.[17, 18] Both the FDA and the American College of Radiology have loosened restrictions on metformin use in updated guidelines.[19, 20] As metformin continuation after insulin initiation has proven benefits, this practice should be encouraged.

The high continuation rates of diabetes medications other than metformin after the transition to insulin were consistent with the previous study on this topic.[12] High rates of continuation were particularly surprising for insulin secretagogues. Use of sulfonylureas or thiazolidinediones in combination with insulin may cause more hypoglycemic episodes and greater weight gain.[11, 21] However, practice guidelines are unclear on whether these medications should be continued when initiating insulin.[7] Given how frequently these diabetes medications are prescribed, and how often they are continued through the insulin transition, it will be important for practice guidelines to provide more specific recommendations as to whether, and under which circumstances, they should be continued after insulin initiation.

We also found that newer diabetes medications (DPP4 inhibitors, GLP-1 receptor agonists, and SGLT2 inhibitors) were all continued in the vast majority of participants initiating insulin. While these diabetes medications have a lower risk of hypoglycemia compared to insulin secretagogues when used as add-on therapy after metformin, there is less evidence for their comparative effectiveness and safety when used with insulin.[22] The findings in this study highlight the need for further research into the use of newer diabetes medications in combination with insulin. In addition, it should be noted that a substantial proportion of the rising costs of diabetes care in the U.S. is attributed to insulin, and newer diabetes medications are also quite costly.[5, 23] Determining the incremental benefit of continuing newer diabetes medication when starting insulin will be important for achieving high value care of patients with type 2 diabetes.

The study’s use of a large, nationally representative data source and broad eligibility criteria provides results that are generalizable to commercially insured adults with type 2 diabetes in the United States. One major strength is the definition of discontinuation that allows flexible regimen adjustments with gaps of up to 90 days which accounts for variations in refill schedules and patient adherence to treatment. However, the study does have several limitations. First, as in all studies using prescription records, participants may not have used the medications that they filled. Second, potential confounders such as blood glucose levels or body weight were unavailable in the administrative claims database. Third, the use of ICD-9 diagnosis codes during the 90 days prior to the index date to exclude patients with type 1 diabetes is imprecise since some patients may not have had a healthcare encounter during that period. Lastly, the use patterns are based on prescriptions paid for by the patient’s insurance plan and do not include prescription issued through discount and loyalty programs that offer several diabetes medications at free or nominal costs. Though our large sample size represented a majority of insured type 2 diabetes patients in the U.S., interpretations of the results should be cautiously generalized to populations with similar sociodemographic characteristics due to the nature of the data source.

## Conclusion

Our analyses provide new insights into the use of diabetes medications after insulin initiation among commercially insured U.S. adults, calling for future studies to expand examinations to a more general population. Clinicians are highly adherent to the current treatment guidelines, which only address the use of metformin following the transition to insulin. The frequent continuation of other diabetes medications while initiating insulin therapy underscores the need for further research into diabetes treatment during the insulin transition to inform evidence-based guidelines that promote optimal management during this critical transition in care.

## Supporting information

S1 Table The non-insulin diabetes medications (Generic name). (DOCX)

S2 Table Non-insulin diabetes monotherapy and combination therapy at baseline (N = 72,971). (DOCX)

S3 Table Rates of treatment continuation, sensitivity analysis based on 2 refills as the marker for medication continuation. (DOCX)

S4 Table Rates of treatment continuation, sensitivity analysis allowing drug in-hand on index date to count as marker for continuation. (DOCX)
