# Supplementary material for: Use of non-insulin diabetes medicines after insulin initiation: A retrospective cohort study
Source: PLoS One. 2019 Feb 13;14(2):e0211820. doi: 10.1371/journal.pone.0211820 (PMC6373953; doi:10.1371/journal.pone.0211820)
Supplement: S1 Table — (DOCX) [file pone.0211820.s001.docx]

**S1 Table. The Non-Insulin Diabetes Medications (Generic Name).**

| **Drug Class** | **Agent (Generic Name)** |
| --- | --- |
| Metformin | Metformin Hydrochloride/Saxagliptin Hydrochloride |
|  | Metformin Hydrochloride/Sitagliptin Phosphate |
|  | Metformin Hydrochloride/Rosiglitazone Maleate |
|  | Metformin Hydrochloride |
|  | Glyburide/Metformin Hydrochloride |
|  | Glipizide/Metformin Hydrochloride |
|  | Metformin Hydrochloride/Pioglitazone Hydrochloride |
|  | Metformin Hydrochloride/Repaglinide |
|  | Dapagliflozin Propanediol/Metformin Hydrochloride |
|  | Linagliptin/Metformin Hydrochloride |
|  | Empagliflozin/Metformin Hydrochloride |
|  | Alogliptin Benzoate/metformin Hydrochloride |
|  | Canagliflozin/Metformin Hydrochloride |
|  | Metformin Hcl;medical Food |
| Sulfonylurea | Chlorpropamide |
|  | Tolazamide |
|  | Tolbutamide |
|  | Glimepiride/Rosiglitazone Maleate |
|  | Glyburide |
|  | Glyburide, Micronized |
|  | Tolbutamide Sodium |
|  | Glimepiride |
|  | Glipizide |
|  | Glyburide/Metformin Hydrochloride |
|  | Glipizide/Metformin Hydrochloride |
|  | Glimepiride/Pioglitazone Hydrochloride |
| Dipeptidyl peptidase 4 inhibitor (DPP4) | Saxagliptin Hydrochloride |
|  | Metformin Hydrochloride/Saxagliptin Hydrochloride |
|  | Metformin Hydrochloride/Sitagliptin Phosphate |
|  | Sitagliptin Phosphate |
|  | Simvastatin/Sitagliptin Phosphate |
|  | Linagliptin |
|  | Linagliptin/Metformin Hydrochloride |
|  | Empagliflozin/Linagliptin |
|  | Alogliptin Benzoate |
|  | Alogliptin Benzoate/metformin Hydrochloride |
|  | Alogliptin Benzoate/pioglitazone Hydrochloride |
| Glucagon-like peptide-1 receptor agonist (GLP1) | Dulaglutide |
|  | Liraglutide |
|  | Albiglutide |
|  | Exenatide |
| Sodium glucose co-transporter inhibitor (SGLT2) | Dapagliflozin Propanediol |
|  | Dapagliflozin Propanediol/Metformin Hydrochloride |
|  | Empagliflozin |
|  | Empagliflozin/Metformin Hydrochloride |
|  | Empagliflozin/Linagliptin |
|  | Canagliflozin |
|  | Canagliflozin/Metformin Hydrochloride |
| Thiazolidinedione (TZD) | Glimepiride/Rosiglitazone Maleate |
|  | Metformin Hydrochloride/Rosiglitazone Maleate |
|  | Rosiglitazone Maleate |
|  | Pioglitazone Hydrochloride |
|  | Metformin Hydrochloride/Pioglitazone Hydrochloride |
|  | Glimepiride/Pioglitazone Hydrochloride |
|  | Alogliptin Benzoate/pioglitazone Hydrochloride |
