# Supplementary material for: Use of non-insulin diabetes medicines after insulin initiation: A retrospective cohort study
Source: PLoS One. 2019 Feb 13;14(2):e0211820. doi: 10.1371/journal.pone.0211820 (PMC6373953; doi:10.1371/journal.pone.0211820)
Supplement: S2 Table — (DOCX) [file pone.0211820.s002.docx]

**S2 Table. Non-Insulin Diabetes Monotherapy and Combination Therapy at Baseline (N=72,971)** ^a^

| **Monotherapy**, n (%) |  |
| --- | --- |
| Metformin | 29,764 (40.1) |
| Sulfonylurea | 12,767 (17.5) |
| Dipeptidyl peptidase 4 inhibitor | 4,097 (5.6) |
| Glucagon-like peptide-1 receptor agonist | 3,525 (4.8) |
| Sodium glucose co-transporter inhibitor | 622 (0.9) |
| Thiazolidinedione | 3,297 (4.5) |
|  |  |
| **Combination therapy**, n (%) |  |
| Metformin plus Sulfonylurea | 9,691 (13.3) |
| Metformin plus Thiazolidinedione | 2,383 (3.3) |
| Other combination ^b^ | 6,825 (9.4) |

a Participants (N=72,971) were exclusively divided into monotherapy users (n=67,505, 74.1%) and combination therapy users (n=18,899, 25.9%). b Other combination consisted of two or two more components including metformin, sulfonylurea, Dipeptidyl peptidase 4 inhibitor, Glucagon-like peptide-1 receptor agonist, Sodium glucose co-transporter inhibitor and Thiazolidinedione.
