# Supplementary material for: Use of non-insulin diabetes medicines after insulin initiation: A retrospective cohort study
Source: PLoS One. 2019 Feb 13;14(2):e0211820. doi: 10.1371/journal.pone.0211820 (PMC6373953; doi:10.1371/journal.pone.0211820)
Supplement: S3 Table — (DOCX) [file pone.0211820.s003.docx]

**S3 Table. Rates of Treatment Continuation, Sensitivity Analysis based on 2 refills as the Marker for Medication Continuation**

| **Treatment Use** | **90 days Before** | **90 days After** | **Continuation (%)** ^a^ |
| --- | --- | --- | --- |
| **Overall, n** | 65,902 | 38,045 | 57.7 |
|  |  |  |  |
| **Drug class, n (%)** |  |  |  |
| Metformin | 47,846 (72.6) | 26,744 (70.4) | 56.0 |
| Sulfonylurea | 22,834 (34.6) | 10,795 (28.4) | 47.3 |
| Dipeptidyl peptidase 4 inhibitor | 7,673 (11.6) | 3,959 (10.4) | 51.6 |
| Glucagon-like peptide-1 receptor agonist | 5,512   (8.4) | 2,847   (7.5) | 51.7 |
| Sodium glucose co-transporter inhibitor | 1,047   (1.6) | 659   (1.7) | 62.9 |
| Thiazolidinedione | 6,789 (10.3) | 3,377  (8.9) | 49.7 |

a : Continuation (%) was defined as a medication was used both before and after insulin initiation.
