# Supplementary material for: Use of non-insulin diabetes medicines after insulin initiation: A retrospective cohort study
Source: PLoS One. 2019 Feb 13;14(2):e0211820. doi: 10.1371/journal.pone.0211820 (PMC6373953; doi:10.1371/journal.pone.0211820)
Supplement: S4 Table — (DOCX) [file pone.0211820.s004.docx]

**S4 Table. Rates of Treatment Continuation, Sensitivity Analysis allowing drug in-hand on index date to count as marker for continuation.**

| **Non-insulin Diabetes Treatment** | Before N (%) | **Excluding patients only with carry-on drug** | | **Including patients only with carry-on drug** | |
| --- | --- | --- | --- | --- | --- |
|  |  | After N (%) | Continuation (%) | After N (%) | Continuation (%) |
| **Overall** | 65,902 | 55,839 | 84.7 | 65,144 | 98.8 |
|  |  |  |  |  |  |
| **Drug class** |  |  |  |  |  |
| Metformin | 47,846 (72.6) | 40,468 (72.5) | 84.6 | 45,980 (70.6) | 96.1 |
| Sulfonylurea | 22,834 (34.6) | 16,815 (30.1) | 73.6 | 21,475 (33.0) | 94.0 |
| DPP4 | 7,673 (11.6) | 6,006 (10.8) | 78.3 | 7,168 (11.0) | 93.4 |
| GLP1 | 5,512 (8.4) | 4,288 (7.7) | 77.8 | 5,105 (7.8) | 92.6 |
| SGLT2 | 1,047 (1.6) | 857 (1.5) | 81.9 | 982 (1.5) | 93.8 |
| Thiazolidinedione | 6,789 (10.3) | 5,386 (9.6) | 79.3 | 6,419 (9.9) | 94.6 |

Abbreviation: DPP4=Dipeptidyl peptidase 4 inhibitor, GLP1= Glucagon-like peptide-1 receptor agonist, SGLT2= Sodium glucose co-transporter inhibitor.
